# Research policy for people with multiple long-term conditions and their carers

**DOI:** 10.1177/26335565221104407

**Published:** 2022-06-14

**Authors:** Natalie Owen, Leanne Dew, Stuart Logan, Simon Denegri, Lucy C Chappell

**Affiliations:** 1 Science, Research & Evidence Directorate, 4620 Department of Health and Social Care, London, UK; 2 NIHR PenARC, 171002 University of Exeter Medical School, Exeter, UK; 3 171002 The Academy of Medical Sciences, London, UK; 4 School of Life Course Sciences, 171002 King’s College London, London, UK

**Keywords:** Multiple long-term conditions, multimorbidity, research policy, public and patient involvement, coordinated care

## Abstract

People with multiple long-term conditions (MLTC) are a growing population, not only in the United Kingdom but internationally. Health and care systems need to adapt to rise to this challenge. Policymakers need to better understand how medical education and training, and service configuration and delivery should change to meet the needs of people with MLTC and their carers. A series of workshops with people with MLTC and carers across the life-course identified areas of unmet need including the impact of stigma; poorly coordinated care designed around single conditions; inadequate communication and consultations that focus on clinical outcomes rather than patient-oriented goals and imperfectly integrate mental and physical wellbeing. Research which embeds the patient voice at its centre, from inception to implementation, can provide the evidence to drive the change to patient-centred, coordinated care. This should not only improve the lives of people living with MLTC and their carers but also create a health and care system which is more effective and efficient. The challenge of MLTC needs to be bought to the fore and it will require joint effort by policymakers, practitioners, systems leaders, educators, the third sector and those living with MLTC to design a health and care system from the perspective of patients and carers, and provide practitioners with the skills and tools needed to provide the highest quality care.

## Introduction

For decades, the health service has largely been shaped around the needs of the system, and healthcare professionals. There is a growing realisation that the health and care system may not work for an increasingly prominent group of patients, those with multiple long-term conditions (MLTC) and that it does not always recognise or respond to the issues that matter most to this group. MLTC presents a complex challenge to policymakers in government and within the health and care systems and allied organisations. If policymakers, healthcare professionals and researchers are to respond effectively and efficiently to the needs of people with MLTC and carers, their voices must be at the heart of any approach. We demonstrate how listening to and involving those with lived experience will enable researchers and policymakers to see these challenges from a different viewpoint.

This paper aims to outline the challenges that people with MLTC and their carers face within the health and care system and how they would like these addressed, drawing on a series of workshops commissioned by the National Institute for Health and Care Research (NIHR) in England^1-3^ and a rapid evidence review of the literature.^4^ Themes emerged from the workshops and then further evidence for their importance and relevant interventions were explored through the rapid review. The paper considers how research co-produced with people with lived experience can provide solutions to change the organisation and delivery of care and where further research is needed to tackle these challenges. Drawing on the experiences of the health and care system described by people with MLTC and their carers, we identified three main areas where change driven by evidence-informed policy could improve services and reduce unmet needs.

## Methodology

Between July and October 2019, three separate workshops were held with carers of children with complex care needs; young, working age and older people with MLTC and carers of older people with MLTC. The methodology across the workshops varied slightly but was designed to ask participants a range of questions to prompt discussion of their experience of services and what mattered to them, including:

• What are your positive/negative experiences of services (health, social care and education)?
• What matters to you?
• What affects you a lot that does not ever get addressed?
• What change would make the biggest difference for you?
• What do we need to learn more about?

The data from the workshops were mapped, synthesised and analysed thematically using framework analysis. Several clear themes emerged: uncoordinated care; person-centred care and empowerment; mental and emotional wellbeing and social isolation; stigma and better understanding of the science behind MLTC. Under each of the themes, participants clearly articulated what they wanted to see change to improve their quality of life, including improved understanding of the system changes needed addressed through research.

The report commissioned on needs for older people also included further examination of evidence from a James Lind Alliance Priority Setting Partnership on Multiple Conditions in Later Life.^5^ Further information about the methodology for these workshops can be found in their published reports.^1-3^

**Figure 1.**
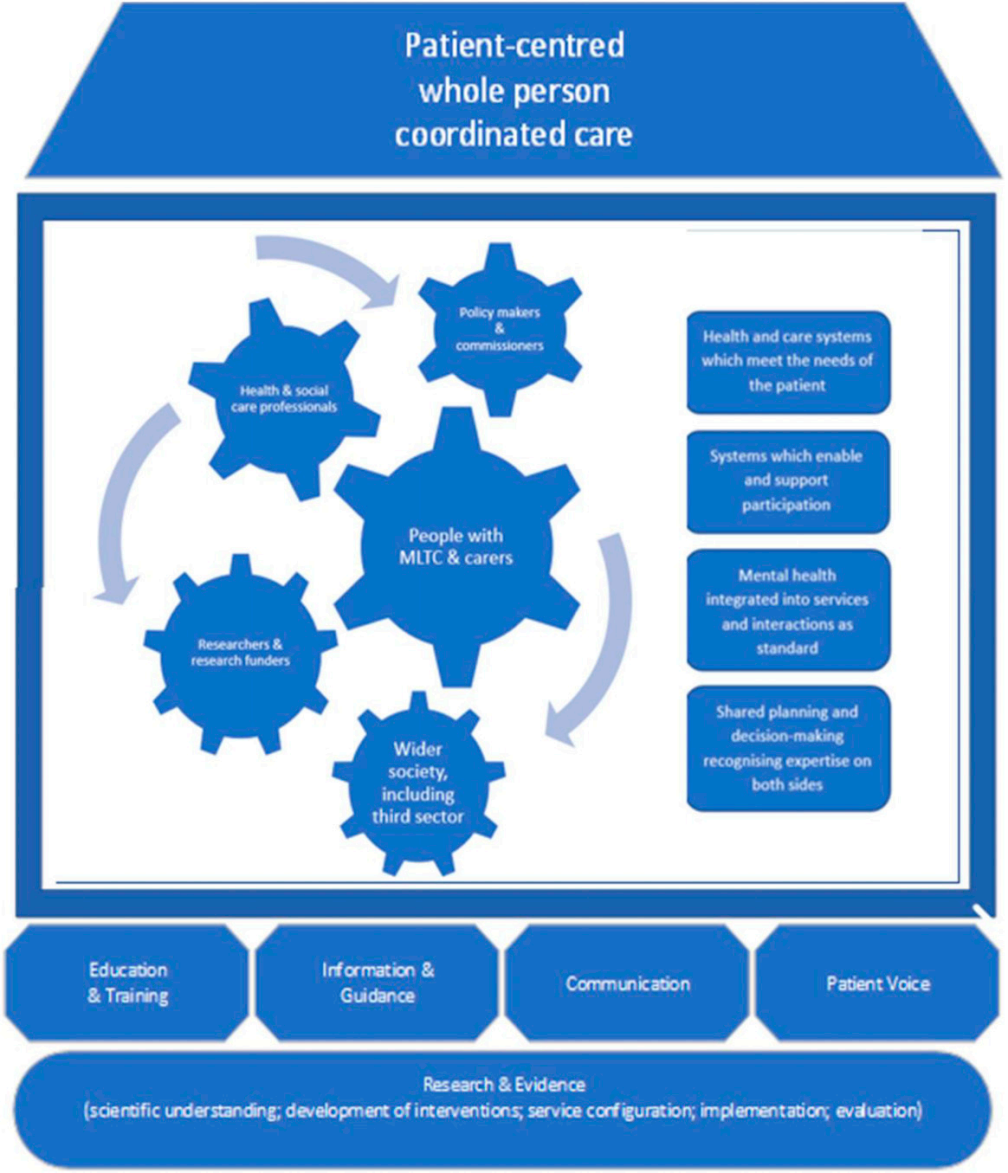
Schematic ‘Message House’ showing the desired outcome (roof) with rectangles inside the house ‘What’) and cogs (‘Who’), underpinned by bricks (‘What needs to be built’) and underpinning foundations (Research and Evidence).

This paper aims to provide an impetus for policymakers, healthcare professionals, commissioners and researchers to discuss how research can inform and drive changes in the design, organisation and provision of health and care services. Below we set out three key challenges arising from the themes identified, backed by a rapid evidence review in each of the areas and show how research can underpin policy transformation across the health and care systems to meet the needs of people with MLTC and carers (Table 1). There remains a gap between the unmet needs expressed at the workshops and application of research policy to address these. Research will not hold all the solutions, but combined with education and training, information and guidance, good communication and a strong patient voice from a diverse population, it can provide the foundations on which change can be built (Figure 1).

**Table 1.** Summary of themes from the workshops and proposed directions of change.

Theme	What do we need?	What will this look like?	Facilitated by?	To provide what patients/carers want
Organisation of health services for patients rather than systems	Changes to service delivery across the health and social care system	- System wide change - Different models of providing care centred around patients with MLTC - Supporting self-management	➢ Healthcare professional education – which moves away from specialisms ➢ Integrated records	• One stop clinics with multiple specialists • A care coordinator to navigate the system and advocate for joined up care across specialists and services • Integrated records – accessible by patient and medical specialists • Continuity of care • Signposting and navigational tools following diagnosis, including where to get advice across different services
Person-centred care through empowerment	Interactions and partnerships within models of care	- Strong relationships and partnerships between patients and clinicians - Patient-centred/ recognising patient as expert - Supporting positive risk taking	➢ Technology ➢ Effective communication models	• Patient at heart of interaction • Sustained holistic care with shared planning and decision-making based on what matters to the patient/family • Better information that reflects the complexity of MLTC • Clear packaging on medication • Good listening and communication leading to understanding and appreciation of lived experience
Mental and emotional wellbeing and social isolation	Enabling bidirectional prevention of mental and physical health problems	- Integration of physical and mental health services	➢ Communication/ Effective conversational models ➢ Education and training for clinicians in supporting mental health as standard	• Clinicians who are confident and able to have conversations about mental health • Mental health services offered at regular and appropriate points • Effective and acceptable interventions to reduce social isolation and loneliness
Addressing stigma	Fix the wider system (population/ institution) and empower (rather than blame) the person	- Health service and civil society working together to raise profile - Asset-based approaches - Empowering people with MLTC	➢ Addressing language ➢ Health and care services and professionals which recognise reality of and are set up for people with MLTC	• Better understanding of stigma faced by people with MLTC • Understanding of barriers and facilitators to participation in everyday life and effective interventions to address them • Health literacy to address in schools and workplaces to educate about lived experience

## Challenge 1: Organising health and care services for patients rather than systems

Poorly coordinated care is an often-heard complaint from people with MLTC,^6^ with multiple appointments with multiple professionals where their stories are told multiple times, at the expense of discussing future care or wellbeing. Participants described a series of barriers within the health and care service that impacted on their quality of life. Time spent ‘being a patient’, attempting to obtain test results and chasing information lost between specialists, systems, organisations and services, compresses time for living. Inconsistencies around diagnostic labels, treatment and management across the services are frustrating and confusing.^7^ Organisation of services appears opaque and ever-changing, with failure to coordinate assessments or management, compounding frustration and fatigue and resulting in a perception that access can be ‘more a fight than a right’. There is a burgeoning literature on the treatment burden of people living with MLTC.^6^ Recent findings from the development of two relevant person-centred outcome measures in people with MLTC^8,9^ emphasised why reducing treatment burden is crucial, showing that high levels of burden were associated negatively with quality of life and self-rated health, and positively related with worsening disease over time.

## Next steps: Changing service delivery across health and social care

People with MLTC and carers who took part in the three workshops were clear they want continuity of co-ordinated care, underpinned by appropriate signposting, support to navigate the system and integrated records to join up delivery of care, with access to a wide range of expertise from different professionals. A shift from increasingly specialised, vertically organised medicine, which often fails to meet the needs of this group, will necessitate engagement across disciplines and organisations. The people taking part in the workshops challenged policymakers to work in partnership with them to organise services from the perspective of the user rather than the clinicians. Research with families has led childhood disability services to aspire to provide ‘Family-Centred Care’,^10^ with multi-disciplinary teams working together to plan integrated assessments and management, often using ‘care coordinators’ as a single point of contact.^11^ Research is needed to explain why, despite these aspirations, families report continuing failures to achieve this goal and to explore the potential wider applicability of this approach among people with MLTC. Services delivered by clinicians with specialist expertise but strong generalist skills^12^ are likely to be central to coordinated care for a range of medical conditions, or clusters, as outlined in the Personalised Care Institute^13^ commissioned by NHS England. Medical education policymakers will be key in developing associated training and research which drives the necessary cultural change.

## Challenge 2: Enabling person-centred care through empowerment

Unsurprisingly, people from the workshops saw themselves as individuals, rather than a collection of conditions or symptoms, and want professionals to be cognisant of the needs of their families and carers; holistic care is more than symptom management and test results. Most wanted to be acknowledged and respected as their own ‘expert’ and empowered to manage their own health and care supported by shared decision-making. They reported that often professionals overlooked non-health-related priorities, with everyday aspirations and life goals disregarded.^14^ There is a requirement for sustained holistic care with better information on their conditions and management to allow those with MLTCs to share planning and decision-making.

## Next steps: Partnerships within new models of care

A sustained shift in culture, as well as service organisation, to enable professionals to prioritise the goals defined by those who use services, alongside conventional clinical outcomes, is needed. Shifting the locus of control to the patient could see functional goals prioritised over clinical outcomes. For example, social care has explored models for enabling positive risk-benefit balancing for service users.^15^ The Comprehensive Geriatric Assessment routinely includes multi-disciplinary assessment and problem resolution or management that determines an older person’s medical, functional, psychological and social capability.^16^ To underpin this shift in emphasis, evaluations of interventions and design of services need to give user-defined outcomes at least equal weight to conventional clinical outcomes. Newly developed measures to assess treatment burden for people living with MLTC will support this endeavour.^8,9^

These models can be enhanced by promotion of self-management, with the patient, or patient and carer, being empowered to take control of their health and wellbeing.^17^ Healthcare technology can be an enabler of person-centred care. Increasing provision of good quality bidirectional information using technology (e.g. from clinicians around treatment and from patients on home monitoring parameters) could improve the range and variety of information exchanged to enhance shared decision-making. Integrated records, held by both clinicians and patient, as already happens in maternity and renal care, could help to facilitate this. There will be substantial lessons from the way that technology has been used during the lockdown phases of the COVID-19 response but it is already apparent from the literature evaluating telemedicine in primary care that we should better understand the barriers to this evolution,^18^ with possible impact on goals such as personalised care, and on unintended consequences such as practitioner workload.^19-21^ These approaches need careful evaluation to ensure that they meet the needs of service users with MLTC rather than the needs of services, and that they do not further increase health inequalities and entrench the Inverse Care Law, ensuring access to and availability of high quality, personalised care for all, especially currently under-served communities living with the highest burden from MLTC.^22^ There is a need to understand for whom digital and technological approaches do not work (or which aspects of care are best managed with other approaches), as well as how they can best be implemented for those for whom they are an effective way to provide care or other management.

## Challenge 3: Incorporating mental and emotional wellbeing into healthcare consultations, and reducing social isolation and stigma

Workshop participants articulated clear needs around their mental and emotional wellbeing. They described often feeling unable to raise these issues during consultations and, when they try, clinicians sometimes find it difficult to respond. Repeated contacts with a sometimes unresponsive health and social care system add stress. People with MLTC talk about a process of loss and grief (whether as a patient or carer) for which they would appreciate expert support to better manage the feelings associated with an enforced change in their vocational, social and domestic abilities, prospects and independence,^23,24^ not to mention financial worries. Social isolation was highlighted by people with MLTC and carers of all ages across all three workshops. They would like healthcare professionals to be better equipped to integrate mental health into all consultations, with social isolation and loneliness openly addressed.^1-3^

Participants spanning the life-course reported stigma and misunderstanding of MLTC in home, school and work settings which left them feeling that society lacks awareness of what it means to live with multimorbidity or complex care needs. Recent research has shown that people with MLTC who experience consistently high treatment burden report more interpersonal challenges with others about their healthcare compared with those with lower treatment burden, suggesting a tension between the people with MLTC and their social networks. This points to a possible misunderstanding of their lived experience.^25^ Many environments fail to accommodate a combination of complexity and nuance, and there can be open hostility, especially for children with behavioural difficulties. People with MLTC said they wanted a better understanding of what drives stigma, and interventions to reduce barriers to participation in everyday life.

## Next steps: Enabling bidirectional prevention of mental and physical health problems and moves to address stigma in a wider system

Services need to address the interaction of physical and mental health proactively from diagnosis and beyond. As individuals age, their conditions change and their needs alter. As an example, the IMPARTS programme explores mental health presentations seen in physical healthcare settings using patient-reported data captured ahead of the first meeting to guide the consultation and treatment plan.^26^ A study of people entering a neuro-otology clinic found that only 5% of those asked to complete a screening tool for common mental health problems were unwilling to do so.^27^ This study and others using the IMPARTS screening tools have examined the prevalence of mental health problems in people with a range of long-term conditions and the role of perceived disease severity in this equation.^28,29^ A number of groups have explored the feasibility and practicability of tailoring diabetes management interventions for persons with learning disability,^30^ autism^31^ or severe mental illness,^32^ recognising the interaction between the conditions and how it impacts treatment.

Innovative conversational models have been proposed that ask the healthcare professional and patient to undertake three steps: sharing problems, linking problems and planning together to address the particular needs of patients with MLTC.^33^ Such projects need embedded process evaluations to identify the components that deliver most, including the mental health and wellbeing outcomes that matter to patients. We need evaluation of models to deliver integrated physical and mental health management and a commitment to make them a key part of future health and care system configuration. These approaches will need professionals for whom the inter-dependency of mental and physical health has been a core concept from the beginning of healthcare education.

The stigma felt by people and families taking part in the workshops who were dealing with complex care needs cannot be addressed by the health and social care system alone. Acknowledging stigma can begin the process of addressing associated poor outcomes including bringing people together to learn about lived experience. For example, the UK Government 2014 strategy, *Think Autism*,^34^ used an integrated approach to policy development to create partnerships across government departments, with people with autism and their families and related charities; it recognised that the challenges required action across society. Lessons from this collaborative approach could be leveraged to increase visibility and reduce stigma for people with MLTC and to develop interventions to facilitate participation.

There is a need to evaluate whether asset-based approaches, which focus on ‘what is strong rather than what is wrong’,^35^ could help to address stigma. Asset-based approaches recognise people as experts in their situation with capacity, skills and knowledge, and practitioners as partners whose theoretical and technical knowledge help them apply these. There are examples of asset-based integrated care models in the literature bringing together primary care, social care, welfare, employment and community services to understand and direct people to the services they need.^36,37^

## Using research to underpin policy developments for MLTC

Large UK research funders, in consultation with single disease research charities, have come together to recognise the overarching evidence needs and cultural changes required within the research system in order to fund the highest quality research in this area.^38,39^ In addition to transformation of the health and care system to meet the needs of people with MLTC, there is growing interest in understanding how conditions interact and cluster, how wider determinants affects the course of a disease and the interplay between physical and mental health conditions. Research on clusters of MLTC may point to aetiological pathways and opportunities for prevention and may indicate how specialisms come together to provide a clinical service that responds to patients as a whole person.^40^ These ambitions also require industry partners to move from a single disease pathway approach for drug and diagnostics development to a broader paradigm.

There is a recognition that future studies of interventions must include people living with MLTC to avoid limiting their applicability; the focus should move from clinical endpoints towards outcome measures such as quality of life, quality of care and treatment burden for both people living with MLTC and carers. These are what matters to patients. We also need to understand how treatments may work optimally, or differently, in those with MLTC and how prevention strategies should factor in MLTC for achievable, realistic outcomes.^41,42^

In order to meet the research aims set out, funders are beginning to understand they need policies to support the development of sustainable career pathway for MLTC researchers, including incentivising experts in single conditions or specialisms to apply their skills to MLTC research. Future funding policy for research on MLTC needs embedded patient involvement from conception to delivery to strengthen the pathway to implementation which should be clear from the start. The resulting evidence will allow policymakers to plan, develop and deliver appropriate healthcare, public health and social care services.

## Conclusion

There is a genuine demand and desire for those commissioning and conducting research and those providing services to work in partnership with people with MLTC and carers. Research funders need to be bolder and consider how their current funding mechanisms can move beyond existing paradigms and shift researchers to think differently, and they need to work with policymakers and practitioners to ensure there is a pull through of research findings into practice. It is not enough to ask the health and care system to change and carry out research to see if it works; we need to use research to drive change, with a more iterative and dynamic approach. The phenomenal response to the COVID-19 pandemic across the world has taught us that policymakers and system leaders can adapt, and we should look to sustainable ways of working that are effective for patients. MLTC should be everyone’s business; a coordinated and coherent plan for action which brings together central and local government, health and care systems, educators, researchers, the third sector and wider society, and has patients and carers in the centre as equal partners, is urgently needed.
